# Short-Term Summer Inundation as a Measure to Counteract Acidification in Rich Fens

**DOI:** 10.1371/journal.pone.0144006

**Published:** 2015-12-04

**Authors:** Ivan S. Mettrop, Casper Cusell, Annemieke M. Kooijman, Leon P. M. Lamers

**Affiliations:** 1 Institute for Biodiversity and Ecosystem Dynamics (IBED), University of Amsterdam, P.O. Box 94248, NL-1090 GE, Amsterdam, The Netherlands; 2 Department of Aquatic Ecology & Environmental Biology, Institute for Water and Wetland Research, Radboud University Nijmegen, NL-6525, Nijmegen, The Netherlands; West Chester University of Pennsylvania, UNITED STATES

## Abstract

In regions with intensive agriculture, water level fluctuation in wetlands has generally become constricted within narrow limits. Water authorities are, however, considering the re-establishment of fluctuating water levels as a management tool in biodiverse, base-rich fens (‘rich fens’). This includes temporary inundation with surface water from ditches, which may play an important role in counteracting acidification in order to conserve and restore biodiversity. Inundation may result in an increased acid neutralizing capacity (ANC) for two reasons: infiltration of base-rich inundation water into peat soils, and microbial alkalinity generation under anaerobic conditions. The main objectives of this study were to test whether short-term (2 weeks) summer inundation is more effective than short-term winter inundation to restore the ANC in the upper 10 cm of non-floating peat soils, and to explain potential differences. Large-scale field experiments were conducted for five years in base-rich fens and *Sphagnum*-dominated poor fens. Winter inundation did not result in increased porewater ANC, because infiltration was inhibited in the waterlogged peat and evapotranspiration rates were relatively low. Also, low temperatures limit microbial alkalinity generation. In summer, however, when temperature and evapotranspiration rates are higher, inundation resulted in increased porewater Ca and HCO_3_^-^ concentrations, but only in areas with characteristic rich fen bryophytes. This increase was not only due to stronger infiltration into the soil, but also to higher microbial alkalinity generation under anaerobic conditions. In contrast, porewater ANC did not increase in *Sphagnum*-plots as a result of the ability of *Sphagnum* spp. to acidify their environment. In both rich and poor fens, flooding-induced P-mobilization remained sufficiently low to safeguard P-limited vegetation. NO_3_^-^ and NH_4_^+^ dynamics showed no considerable changes either. In conclusion, short-term summer inundation with base-rich and nutrient-poor surface water is considered beneficial in the management of non-floating rich fens, and much more effective than winter inundation.

## Introduction

Rich fens are minerotrophic peatland habitats that are characterised by base-rich and nutrient-poor conditions [1]. In Europe, these biodiverse rich fens have become very rare, mainly due to changes in land use, acidification and eutrophication [2], and are therefore protected as EU priority habitat *H7140—Transition mires and quaking bogs*. In terms of conservation and restoration of rich fens, conditions must be base-rich and nutrient-poor to prevent transformation of these species-rich communities to species-poor *Sphagnum*-dominated communities [3].

The cause of the problem of acidification lies in several processes. Hydrological isolation from base-rich groundwater and surface water, caused by natural succession and/or anthropogenic intervention, has led to reduced acid neutralizing capacity (ANC) in fen peatland regions with intensive agriculture [4, 5]. Presumably, increased atmospheric N-deposition as a result of fossil fuel combustion and intensive cattle farming has exacerbated the acidification of fens due to direct influx of nitric acid, and indirectly by additional ammonium oxidation during periods of drought [6, 7]. In addition, P-eutrophication may lead to rapid succession in rich fens, and hence a shift from minerotrophic bryophytes to *Sphagnum* spp. [8]. As these *Sphagnum* spp. release protons in exchange for other cations [9, 10], acidification of the bryophyte layer is indirectly intensified via P-eutrophication.

During the past decades, water levels in European rich fen areas have often been constricted within narrow limits as a result of adjacent agricultural water management. Water authorities are, however, considering the re-establishment of a more natural water regime in these areas, in which temporary inundation with surface water may play an important role in counteracting acidification. Short-term inundation with base-rich water has been postulated as a measure to restore the ANC in the top-soil of fens that lack sufficient HCO_3_^-^ and Ca buffering [11]. Several studies have focused on potential benefits and drawbacks of raised water levels in fens. Winter inundation by raising surface water levels in the field did not result in enhanced ANC in non-floating rich fens [12]. However, during an unexpected wet period in summer, alkalinization did increase, which suggested that inundation may be more effective as a measure in summer than in winter, and further research was suggested to gain a better understanding of the impact of seasons [12]. Two explanatory mechanisms were proposed to explain the differential affects between summer and winter inundation [13]. The primary explanation lies in infiltration of HCO_3_^-^ and Ca-rich inundation water into the peat soil. This is important, since especially Ca-input may contribute to a more permanent increase in ANC, as this is not only determined by the concentration of bicarbonate in porewater, but also by the amount of Ca attached to the adsorption complex [14]. In winter, these infiltration rates may be limited in the already waterlogged soils. In summer, when temperatures are higher, infiltration of base-rich inundation water is presumably facilitated by high evapotranspiration rates [12]. In addition, porewater ANC may increase under anaerobic conditions by reduction of NO_3_^-^, Fe(III) and/or SO_4_, which are processes that lead to microbial alkalinity generation [14, 15]. This microbial alkalinity production may be higher in summer as well, since increased temperatures generally promote microbial activity in anaerobic peat soils. In contrast to Ca supply via infiltration, the effect of internal alkalinization may be temporary, because subsequent aeration of the peat soil may lead to oxidation-induced acidification [16, 17]. In addition, alkalinity that is not trapped as, or adsorbed onto, a solid state during reduction can potentially migrate away from the inundated site by surface overflow [18].

Besides the positive effects of inundation with base-rich water on the ANC of fen soils, also potential adverse effects have to be taken into account. Anaerobic conditions may result in net P-mobilization (internal eutrophication) due to Fe reduction [19], potentially further increased by SO_4_ reduction [20], and hence increased P-availability [13, 17, 21]. In addition, anaerobic conditions may lead to formation of potential phytotoxins such as NH_4_^+^, H_2_S, Fe^2+^ and/or organic acids [2]. All of these potential adverse effects may be stimulated by inundation and need to be assessed as well.

The main objective of this study was to assess the effectiveness of short-term (2 weeks) summer inundation versus short-term winter inundation to restore ANC in the upper 10 cm of non-floating peat soils. Large-scale field experiments were conducted for several years in base-rich fens and *Sphagnum*-dominated poor fens. We expected short-term inundation with base-rich water to be much more effective in summer than in winter, primarily because relatively high summer temperatures may result in accelerated infiltration of base-rich inundation water, and additionally in accelerated microbial alkalinity generation. Presumably, improvement of the ANC is stronger in base-rich fens than in *Sphagnum*-dominated fens, due to the ability of *Sphagnum* to acidify its environment [9, 10, 12]. Furthermore, we expected limited internal P-mobilization and limited formation of toxins during short-term inundation of two weeks under summer conditions, based on results from earlier field studies in the same area [12] and laboratory mesocosm experiments involving soils from these fen sites [13].

The results of this study are important in relation to the conservation and restoration of endangered rich fen habitats. Increased understanding of biogeochemical processes upon inundation is essential to support water and nature management authorities in environmental decision making, as it explains under which particular conditions restoration measures are expected to be successful. In addition, our results have important additional implications for future management in the face of climate change, since short-term extreme weather events, such as summer flooding, are predicted to occur more frequently [22, 23].

## Material and Methods

### Field site and experimental setup

The field experiments were conducted in two non-floating fens in the Dutch National Park Weerribben-Wieden: ‘Kiersche Wiede’ (KW; 52°41'49.1"N 6°07'56.7"E) and ‘Veldweg’ (VW; 52°41'30"N 6°06'45"E). Natuurmonumenten and Water Management Authority Reest & Wieden are acknowledged for permission and cooperation in these fens. We confirm that no endangered or protected species were sampled during the experiments.

Both fens comprised three vegetation types: (1) labelled ‘Scor’: rich fens with respectively *Hamatocaulis vernicosus* (Mitt.) Hedenäs (*Caricion nigrae*–*Carex nigra*-*Agrostis canina* type) in KW and *Scorpidium cossonii* (Schimp.) Hedenäs (*Caricion davallianae*–*Scorpidium*-*Carex diandra* type) in VW, (2) ‘Call’: rich fens with *Calliergonella cuspidata* (Hedw.) Loeske dominating the moss layer (*Caricion nigrae*–*Carex nigra*-*Agrostis canina* type), and (3) ‘Sph’: poor fens with *Sphagnum palustre* L. and *Sphagnum fallax* (H.) Klinggr. dominating the moss layer (*Caricion nigrae*–*Pallavicinio-Sphagnetum typicum* type).

An isolated part (9000 m^2^) of the KW-fen was chosen as experimental site with raised surface water levels, while part of the VW-fen (9375 m^2^) was chosen as a reference site in which the water level remained unchanged. Generally, surface water levels in the area are constricted within 0.73 and 0.83 m below mean sea level (BMSL). Short-term inundation of the fen surface in the KW-fen was achieved by raising the surface water level up to 0.63 BMSL during 14 days by using a pump. This meant a raise of 10 cm in November 2009, 2010 and 2011, and 15 cm in July-August 2013 and 2014. In both fens, the selected plots were located within a maximum distance of 50 m from adjacent ditches, and none of the fens was floating due to root attachment to the sand substrate at a depth of 60–90 cm.

### Sampling and analyses

For each vegetation type (Scor, Call and Sph), five plots were selected in both KW and VW (*n*_tot_ = 30). All measurements were carried out (a) 2 days before, (b) halfway during, and (c) 2 days after experimental manipulation of the surface water level. Water tables in the fen soils were manually recorded. Porewater samples from the upper 10 cm of the peat soils and from surface water in adjacent ditches were collected by using ceramic soil moisture samplers (Rhizon SMS-10 cm; Eijkelkamp Agrisearch Equipment, The Netherlands), connected to vacuumed plastic syringes of 50 mL. After 1 week of inundation, additional samples of the inundation water were collected, also by using these ceramic soil moisture samplers.

pH-values were measured with a standard Ag/AgCl electrode and alkalinity was determined by titration down to pH 4.2 using 0.01 mol L^-1^ HCl. Concentrations of dissolved o-PO_4_, NO_3_^-^, NH_4_^+^, SO_4_, Cl and dissolved organic matter (DOC) were measured by using auto-analyzer (Skalar, San^++^ System, fitted with Skalar, SA1074). Total concentrations of dissolved Ca, Fe, and S were measured in acidified subsamples by ICP (Perkin-Elmer, Optima 3000XL).

Ca and Cl-concentrations were used to calculate the ionic ratio (IR), which is equal to 2*[Ca]/(2*[Ca]+[Cl]. This IR index can be used as an indicator of the relative influence of groundwater and/or surface water versus rainwater in porewater [4]. The IR is very popular for discriminating between atmocline (precipitation-like) and lithocline (long-residence groundwater-like) water types. Further, porewater ratios of [alkalinity]/[Cl] and [Ca]/[Cl] were used as indicators of infiltration, because of the suitability of Cl as an inert tracer.

### Continuous redox measurements

In all vegetation types, the redox potential (*E*_h_) in the upper 20 cm was measured as a proxy for the extent to which oxygen availability was affected. The *E*_h_ was measured in Call-vegetation during summer inundation in 2013, and in Scor- and Sph-vegetation during summer inundation in 2014. Permanently installed fiberglass probes with platinum sensor tips at different heights (PaleoTerra, Amsterdam, The Netherlands), connected to a Hypnos data logger (MVH Consult, Leiden, The Netherlands) [24] were used to record *E*_m_ (measured potential) at -1 cm, -3 cm, -5 cm, -10 cm, -15 cm, and -20 cm below the soil surface every 15 minutes. *E*_m_ was measured as the potential between a sensor tip and a 3M Ag/AgCl reference probe. The *E*_h_ was calculated by adding a standard reference voltage and correcting for differences in pH, since pH indirectly modifies the Nernstian effect of the redox electrode:

*E*_h_ = *E*_m_ + *E*_ref_− 59 * (7 –pH), with *E*_ref_ being the potential of the reference probe.

### Statistical analysis

Initial differences in water tables and porewater chemistry were tested by a linear mixed model with LSD (least significant difference) post-hoc analyses, using location (KW vs. VW), vegetation type (Scor, Call and Sph) and season (winter vs. summer) as three fixed factors. Initial differences in surface water chemistry were tested with location and season as fixed factors. Since subreplicates were taken consecutively over the years from the same plots, the model was run with ‘AR(1): Heterogeneous’ as residual repeated covariance structure, with year as repeated effect. For all analyses yearly initial values, as measured 2 days before the experiment, were used.

Also for the treatment results a linear mixed model, with year as repeated effect, was used to test the response to three main fixed factors 1) water level treatment: inundation in KW vs. reference in VW, 2) season: winter vs. summer, and 3) vegetation type: Scor, Call and Sph. Differences between measurements before and after surface water level manipulation were used as response variables. In addition, differences in inundation water characteristics in KW were tested, using vegetation type and season as fixed factors. Differences in response between the three vegetation types were further tested by LSD post-hoc analyses.

All statistical analyses were performed using SPSS 20.0 for Windows (IBM Inc., 2011). *P*-values in the text are indicated as follows: *P < 0.05, **P < 0.01.

## Results

### Initial conditions

#### Surface water characteristics

The surface water quality in adjacent ditches differed between the experimental site (KW) and the reference site (VW). Surface water in VW was, with an average Ca concentration of 1200 μmol L^-1^ and an alkalinity of around 2.5 mmol_c_ L^-1^, twice as base-rich as surface water in KW with average Ca concentrations of 630 μmol L^-1^ and an alkalinity of around 1.2 mmol_c_ L^-1^ (*F*_1,7_ = 40.74** and *F*_1,6_ = 24.4**). Neither Ca concentrations, nor alkalinities in surface water differed between seasons (*F*_1,7_ = 0.8^NS^ and *F*_1,9_ = 3.39^NS^). Surface water o-PO_4_ concentrations did not differ between locations (*F*_1,36_ = 0.25^NS^) and were slightly increased in summer (*F*_1,38_ = 68.54**), but still relatively low with values below 1.0 μmol L^-1^. Surface water NO_3_^-^ concentrations were higher in summer than in winter (*F*_1,43_ = 44.73^NS^) and this summer-induced increase was stronger in VW than in KW, as indicated by a significant interaction of location*season (*F*_1,43_ = 18.24**). However, NO_3_^-^ concentrations remained relatively low with average values under 10 μmol L^-1^. Also NH_4_^+^ concentrations turned out to be higher in summer with average concentrations of 13.5 μmol L^-1^ versus 4.0 μmol L^-1^ in winter (*F*_1,44_ = 85.69**), with no difference between the two locations (*F*_1,41_ = 0.47^NS^).

#### Soil porewater characteristics

Initial soil porewater Ca-concentrations, alkalinities and pH were generally lower in KW than in VW (Table 1). This differences are more obvious in the Scor- and Call-plots than in the Sph-plots, as indicated by significant interaction of location*vegetation type (Table 2). However, the ionic ratio (IR) did not differ significantly between the two fen sites, suggesting that the relative influence of base-rich surface water did not differ between KW and VW. Furthermore, water tables in the soil at *T* = 0 did not differ between KW and VW, and overall nutrient concentrations were relatively low in both sites.

**Table 1:** Initial water tables and porewater characteristics for the different areas and vegetation types for combined seasons.

	Scor		Call		Sph		Ditch	
Variable	KW	VW	KW	VW	KW	VW	KW	VW
Water table (cm)	-0.4 (1.0)	-1.1 (1.4)	-3.3 (0.8)	-5.1 (1.0)	-8.5 (1.0)	-10.9 (1.0)	-	-
Cl (μmol L^-1^)	480 (31)	752 (57)	427 (35)	617 (63)	276 (24)	487 (51)	664 (53)	895 (58)
IR (mol mol^-1^)	0.57 (0.01)	0.65 (0.02)	0.63 (0.02)	0.65 (0.02)	0.53 (0.03)	0.52 (0.02)	0.65 (0.01)	0.71 (0.01)
Ca (μmol L^-1^)	315 (15)	723 (63)	358 (20)	509 (35)	163 (15)	230 (16)	583 (45)	1123 (85)
Alkalinity (μmol_c_ L^-1^)	409 (33)	1280 (146)	567 (57)	789 (67)	115 (16)	235 (37)	1160 (99)	2248 (199)
pH	5.7 (0.1)	6.3 (0.1)	5.9 (0.1)	6.0 (0.1)	4.9 (0.1)	5.4 (0.1)	7.0 (0.1)	7.4 (0.1)
Fe (μmol L^-1^)	18.7 (4.0)	14.7 (3.4)	21.1 (7.4)	13.9 (2.5)	39.6 (10.5)	35.1 (6.3)	2.1 (0.3)	3.3 (1.0)
S (μmol L^-1^)	71.1 (15.5)	31.2 (3.7)	80.9 (19.8)	74.3 (11.2)	47.1 (9.3)	43.1 (3.8)	143.7 (7.7)	154.5 (12.3)
o-PO_4_ (μmol L^-1^)	0.51 (0.13)	0.59 (0.11)	0.64 (0.15)	0.75 (0.22)	1.00 (0.20)	1.51 (0.38)	0.44 (0.12)	0.77 (0.32)
NH_4_^+^ (μmol L^-1^)	2.72 (0.40)	4.77 (0.92)	4.49 (1.45)	4.28 (0.59)	5.93 (1.61)	5.09 (0.78)	7.19 (1.99)	8.71 (2.41)
NO_3_^-^ (μmol L^-1^)	1.77 (0.46)	3.71 (0.84)	1.18 (0.17)	3.38 (0.68)	1.72 (0.24)	3.93 (0.93)	2.41 (0.70)	14.32 (6.10)

Data shown represent mean values with S.E. (*n* = 25). Scor = fen dominated by *Scorpidium cossonii* or *Hamatocaulis vernicosus*, Call = fen dominated by *Calliergonella cuspidata*, Sph = fen dominated by *Sphagnum palustre*, Ditch = surface water in adjacent ditch. KW = Kiersche Wiede (experimental fen site), VW = Veldweg (reference fen site). IR (Ionic Ratio) = 2*[Ca]/(2*[Ca]+[Cl]).

**Table 2:** Effects of location, season, vegetation type and their interactions on the water table and porewater chemistry at *T* = 0 of each yearly experiment.

Variable	Location	Season	Veg	Location*Season	Location*Veg	Season*Veg	Scor	Call	Sph
Water table	0.01 (43.3)	0.89 (106.0)	55.24** (43.4)	3.16 (106.0)	0.95 (38.4)	1.19 (106.0)	c	b	a
Cl	52.59** (38.6)	19.86** (43.4)	27.54** (38.6)	5.37* (43.4)	1.57 (49.5)	7.18* (43.4)	b	b	a
IR	3.29 (47.7)	16.90** (65.7)	16.91** (47.6)	1.32 (65.7)	2.82 (49.5)	2.82 (65.7)	b	b	a
Ca	47.05** (38.1)	10.07** (99.7)	39.42** (38.1)	0.13 (99.7)	8.17** (40.0)	2.90 (99.6)	b	b	a
Alkalinity	27.38** (39.2)	0.01 (112.9)	23.45** (39.3)	0.08 (112.7)	7.99** (38.6)	0.86 (112.7)	b	b	a
pH	44.02** (38.6)	3.01 (76.7)	95.34** (38.7)	1.21 (76.5)	4.64* (37.8)	2.41 (76.6)	b	b	a
Fe	2.39 (60.9)	3.11 (68.8)	11.80** (60.9)	3.65 (68.6)	2.30 (39.7)	2.04 (68.6)	a	a	b
S	19.03** (30.8)	46.86** (30.3)	3.81* (30.8)	7.35* (30.4)	3.80* (35.1)	0.69 (30.4)	ab	b	a
o-PO_4_	0.62 (31.8)	87.54** (33.9)	7.28** (31.8)	0.99 (33.9)	0.95 (42.4)	3.00 (33.9)	a	a	b
NH_4_^+^	0.67 (36.5)	98.14** (39.0)	3.33 (36.5)	0.23 (39.0)	0.14 (49.7)	2.01 (39.0)	n.s.	n.s.	n.s.
NO_3_^-^	25.62** (35.9)	40.59** (39.1)	0.58 (35.6)	15.91** (38.9)	1.54 (43.7)	0.15 (38.9)	n.s.	n.s.	n.s.
DOC	2.62 (42.1)	38.02** (72.5)	11.31** (42.3)	3.14 (71.8)	2.85 (45.2)	0.04 (72.1)	a	a	b

For abbreviations see Table 1. *F*-ratios including denominator d.f. in parentheses are shown with their level of significance:

*P < 0.05

**P < 0.01.

Different letters indicate significant differences (*P* < 0.05) between vegetation types. n.s. = not significant.

The initial water tables in the peat soil differed among vegetation types (Tables 1 and 2). Scor-plots were characterized by water tables less than 1 cm beneath the soil surface, while Call-plots showed an average water table of -4 cm. In Sph-plots the water table was even lower with an average level at -10 cm. Furthermore, the influence of base-rich surface water was higher in Scor- and Call-plots than in Sph-plots, as indicated by a higher porewater IR. Consequently, porewater in Scor- and Call-plots showed significantly high alkalinities of about 0.8 and 0.7 mmol_c_ L^-1^, and Ca-concentrations of about 500 and 400 μmol L^-1^, while in Sph-plots initial alkalinities were about 0.2 mmol_c_ L^-1^ and average Ca-concentrations did not exceed 200 μmol L^-1^. As expected, average initial porewater pH values of 6.0 in both Scor- and Call-plots were also significantly higher than in Sph-plots, where pH values of about 5.1 were measured. In contrast, both Fe- and o-PO_4_ concentrations in porewater were two times higher in Sph-plots than in Scor- and Call-plots.

While the initial water tables in the peatsoil did not differ between winter and summer, initial Cl, Ca, S, o-PO_4_, NH_4_^+^, NO_3_^-^ and DOC concentrations in porewater were generally higher in summer. Particularly in VW the initial NO_3_^-^ concentrations were increased in summer, as indicated by a significant interaction of location*season (Table 2).

### Winter inundation vs. summer inundation

#### Inundation and infiltration

**Fig 1:**
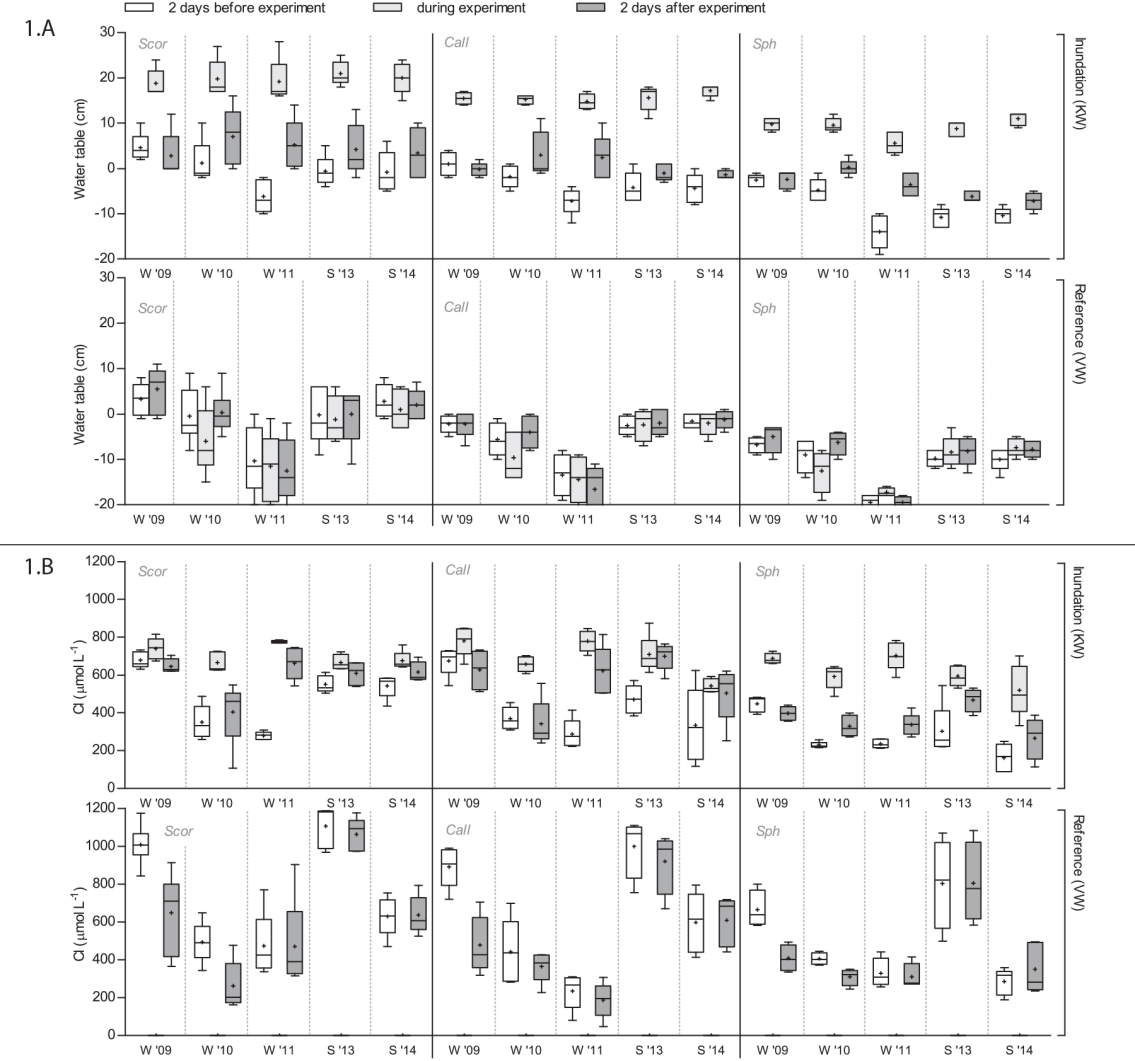
Water table (A) and Cl-concentrations (B) per vegetation type in porewater 2 days before the experiment, in inundation water during the experiment, and in porewater 2 days after the experiment. Sample means with standard deviations are indicated (*n* = 5). Statistical information is provided in Table 3. For abbreviations see Table 1.

Raising of the surface water level in adjacent ditches in KW clearly affected the height of the water tables in the peat soil in all vegetation types (Fig 1A). Both in winter and in summer, the soil surface in all KW-plots became inundated via lateral flow from the ditches, while water tables in the reference location VW did not change. The height of the inundation water level relative to the surface level generally differed among vegetation types in KW, even though in all plots the peat layer was attached to the underlying sand substrate. In Sph-plots the layer of inundation water was two times less thick than in Scor- and Call-plots (*F*_1,21_ = 34.95**), due to the relatively higher position in the landscape of the soil surface in *Sphagnum*-plots.

In the reference site VW without inundations, neither in winter nor in summer there were changes in porewater Cl, which was used as an inert tracer for the rate of infiltration. In the experimental site KW, winter inundations in 2009 and 2010 with Cl-rich surface water from the adjacent ditches did not result in increased Cl-concentrations in porewater either (Fig 1B). This indicates that, despite obvious inundation in all plots, infiltration in the peat soil did not occur. Only in the winter of 2011, when initial water tables in the peat soil were relatively low with levels around -10 cm, porewater Cl-concentrations increased, pointing to actual infiltration of Cl-rich inundation water.

In contrast, inundation during the summers of 2013 and 2014 resulted in a significant increase in both porewater Cl-concentrations and porewater IR, as indicated by an interaction of inundation*season (Table 3). Porewater Cl-concentrations approached 650 μmol L^-1^, which was almost equal to the concentrations in the inundation water, and these changes did not differ among vegetation types. This implies that in all vegetation plots infiltration rates were higher during summer inundation than during winter inundation.

**Table 3:** Effects of inundation, season, vegetation type and their interactions on porewater chemistry during the experiments.

Variable	Inundation	Season	Veg	Scor	Call	Sph	Inundation*Season	Inundation*Veg	Season*Veg
Cl	99.07** (46.3)	42.70** (65.6)	2.10 (46.3)	n.s.	n.s.	n.s.	5.44* (65.7)	3.09 (41.1)	0.92 (65.6)
IR	10.30** (52.3)	0.70 (62.1)	1.01 (52.3)	n.s.	n.s.	n.s.	15.94** (62.1)	0.60 (55.3)	2.08 (62.1)
Ca	33.71** (44.3)	18.17** (93.6)	1.26 (44.3)	n.s.	n.s.	n.s.	3.65* (93.8)	3.53* (44.0)	2.42 (93.7)
Alkalinity	60.83** (45.9)	29.80** (83.5)	4.33* (46.0)	b	ab	a	4.05* (83.4)	16.89** (45.8)	3.30* (83.3)
pH	4.27 (46.8)	0.02 (57.1)	11.04** (46.8)	b	b	a	0.72 (57.0)	1.43 (41.3)	2.39 (57.1)
Fe	2.85 (39.3)	2.32 (43.6)	3.12 (39.2)	n.s.	n.s.	n.s.	0.01 (43.4)	0.84 (57.5)	1.30 (43.6)
S	1.69 (40.3)	0.17 (45.1)	0.83 (40.3)	n.s.	n.s.	n.s.	12.26** (45.0)	0.64 (39.0)	1.15 (45.0)
o-PO_4_	7.68** (40.3)	4.03 (50.7)	0.11 (40.3)	n.s.	n.s.	n.s.	6.35* (50.7)	0.13 (46.0)	0.31 (50.7)
NO_3_^-^	3.46 (50.9)	1.40 (56.7)	0.13 (50.6)	n.s.	n.s.	n.s.	0.22 (56.5)	0.58 (44.0)	2.07 (56.4)
NH_4_^+^	84.19** (26.9)	78.36** (29.2)	3.86* (26.9)	b	a	a	73.3** (29.1)	1.75 (49.0)	3.33 (29.0)
DOC	3.09 (51.2)	23.82** (85.9)	10.92** (51.1)	a	a	b	0.36 (85.7)	5.51** (53.8)	4.52* (85.7)

For abbreviations see Table 1. *F*-ratios including denominator d.f. in parentheses are shown with their level of significance:

*P < 0.05

**P < 0.01.

Different letters indicate significant differences (*P* < 0.05) between vegetation types. n.s. = not significant.

#### Changes in redox potential

Redox potentials (*E*_h_), which were only measured in summer, obviously decreased upon inundation (S1 Fig). Particularly in Scor-vegetation, the peat soil showed constant anaerobic conditions with *E*_h_ values below -200 mV during inundation. In Call-plots, anaerobic conditions were less prevalent, and the upper 5 cm was not characterized by anaerobic circumstances in the first week of inundation. Moreover, in Sph-plots, anaerobic conditions were eliminated after the first three days of inundation, and aerobic conditions with *E*_h_ values of 300–400 mV prevailed during the rest of the inundation period, even though the moss layer was submerged.

#### ANC and pH in porewater

**Fig 2:**
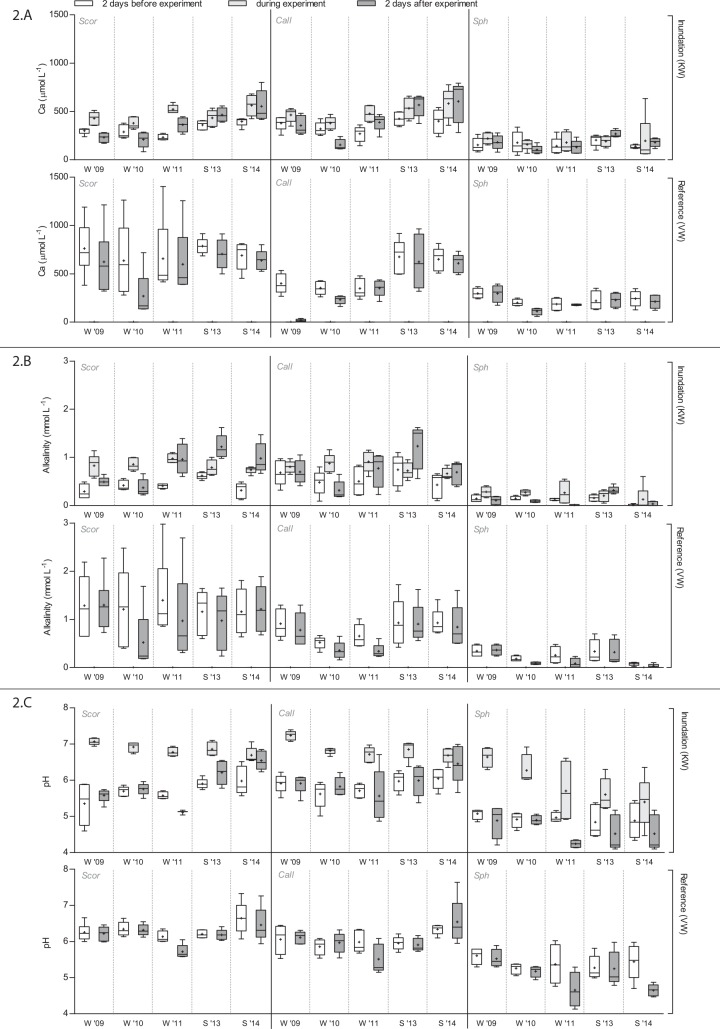
Ca-concentrations (A), alkalinity (B) and pH (C) per vegetation type in porewater 2 days before the experiment, in inundation water during the experiment, and in porewater 2 days after the experiment. Sample means with standard deviations are indicated (*n* = 5). Statistical information is provided in Table 3. For abbreviations see Table 1.

In the reference site VW, there were no changes in porewater Ca-concentrations and alkalinity, neither in winter nor in summer. Winter inundation in KW did not result in increased Ca-concentration or alkalinity in porewater either (Fig 2A and 2B). Just like with Cl-concentrations, infiltration of base-rich surface water resulted in slightly increased porewater Ca-concentrations and alkalinity only in the winter of 2011, when initial water tables were relatively low.

In contrast, summer inundations in KW in 2013 and 2014 resulted in a significant increase in both porewater Ca-concentrations and alkalinity, as indicated by an interaction of inundation*season (Table 3). Ca-concentrations increased with 120 μmol L^-1^ on average, and alkalinities showed an increase of about 0.4 mmol_c_ L^-1^. This increase in porewater ANC during summer inundation did, however, not result in a significant change in porewater pH.

The effect of inundation on the porewater ANC in KW did not only differ between winter and summer, but also among vegetation types. Both Ca-concentrations and alkalinity in porewater showed an increase in the Scor- and Call-plots, but this increase was absent in the Sph-plots, as indicated by an interaction of inundation*vegetation type (Table 3). Also, inundation water during both winter and summer experiments showed much lower Ca-concentrations and lower alkalinities in the Sph-plots (*F*_1,20_ = 70.67** and *F*_1,21_ = 84.14**). As a result, the pH values of inundation water at the Sph-plots were considerably lower than at the Scor- and Call-plots (*F*_1,21_ = 52.53**), which was particularly true during summer as indicated by a significant interaction of vegetation type*season (*F*_1,29_ = 6.98**).

#### Nutrients in porewater

**Fig 3:**
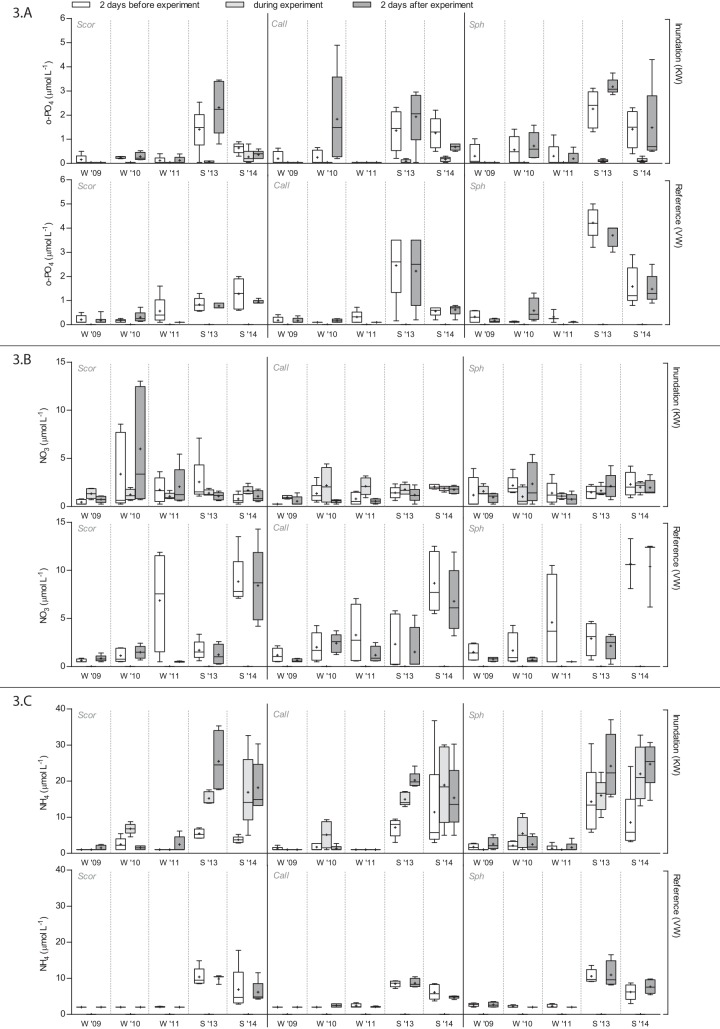
o-PO_4_ (A), NO_3_^-^ (B) and NH_4_^+^ (C) concentrations per vegetation type in porewater 2 days before the experiment, in inundation water during the experiment, and in porewater 2 days after the experiment. Sample means with standard deviations are indicated (*n* = 5). Statistical information is provided in Table 3. For abbreviations see Table 1.

Inundation in KW resulted in a small increase in porewater o-PO_4_ concentrations of 0.3 μmol L^-1^ on average during summer inundation (Fig 3A and Table 3). This increase was only detected in 2013, and was presumably the result of internal P-mobilization, since the o-PO_4_ concentrations in supplied surface water from the adjacent ditch were lower than these concentrations in porewater.

Porewater NO_3_^-^ concentrations were generally unaffected by inundation (Table 3) and remained very low in all vegetation types, regardless of the season (Fig 3B). NH_4_^+^ concentrations in porewater were, however, significantly affected by inundation and this effect differed between seasons (Fig 3C and Table 3). During summer inundation, an average increase of NH_4_^+^ concentrations of 13 μmol L^-1^ was measured, while during winter inundation NH_4_^+^ concentrations remained unaltered and very low.

## Discussion

### Conditions before inundation

Initial differences in pH and ANC between Sph-plots versus Scor- and Call-plots are due to the minor influence of base-rich water in Sph-plots, since *Sphagnum* spp. naturally occur further above the water table, are mainly fed by rain water, and acidify their habitat by releasing protons in exchange for other cations [9]. This reduces pH and ANC along the fen-bog gradient [4, 25]. The lower pH in Sph-plots leads to increased solubility of iron and calcium phosphates, possibly explaining increased o-PO_4_ concentrations in Sph-plots [26].

The overall slightly increased initial porewater Ca- and Cl-concentrations in summer are presumably due to reduced dilution with rainwater due to increased evapotranspiration in summer [4], as confirmed by an increased IR. While Ca- and Cl-concentrations were only 1.2 times higher in summer, o-PO_4_ and NH_4_^+^ concentrations showed a stronger increase of 5–6 times, which we attribute not only to reduced dilution, but moreover to increased net mineralization rates as a result of the higher temperatures. Increased DOC-concentrations, as an indicator of increased decomposition, seem to confirm this idea.

### Summer versus winter inundation

Generally, winter inundation did not result in infiltration of inundation water, because the peat soils were already waterlogged and evapotranspiration rates were relatively low [12]. Only when initial water tables were sufficiently low (about 10 cm below the soil surface), infiltration of base-rich surface water was facilitated in winter. In contrast, inundation in summer, when the peat is less water-saturated and evapotranspiration rates are higher, did result in enhanced infiltration of inundation water rich in Ca and HCO_3_^-^.

Infiltration of base-rich surface water may, however, not have been the only process resulting in an increased porewater ANC upon inundation. To get insight into the relative contribution of internal, microbial alkalinity generation, porewater ratios of [alkalinity]/[Cl] and [Ca]/[Cl] were calculated (Table 4). In Scor-plots the ratio [alkalinity]/[Cl] was more than twice as high after summer inundation, while this was not the case after winter inundation. Further, the ratio [Ca]/[Cl] showed only a slight increase, which did not differ between winter and summer. This indicates a relatively higher production of alkalinity during summer inundation. Since inundation led to anaerobic conditions, as shown by redox-measurements, anaerobic microbially mediated redox processes occur which result in alkalinity generation [14, 15]. The response of redox potentials to summer inundation did not differ from the response to winter inundation [12]. We, however, suggest that especially during summer inundations, when temperatures are higher and microbial activity is enhanced, microbial alkalinity generation increases in the top-soil of Scor-plots. In the top-soil of Call- and Sph-plots, where anaerobic conditions during inundation were less prevalent than in Scor-plots, anaerobic microbial alkalinity generation may have been smaller, possibly explaining different response in porewater ANC among the vegetation types.

**Table 4:** Average porewater ratios of [alkalinity]/[Cl], and [Ca]/[Cl] in winter and summer, before and after inundation.

Vegetation type		Scor	Call	Sph
*[Alkalinity]/[Cl]*				
Winter	Before inundation	1,0	1,3	0,4
	After inundation	1,0	1,0	0,2
Summer	Before inundation	0,8	1,5	0,4
	After inundation	1,8	1,6	0,5
*[Ca]/[Cl]*				
Winter	Before inundation	0,8	0,7	1,1
	After inundation	0,6	0,5	0,9
Summer	Before inundation	0,8	0,8	1,8
	After inundation	1,0	0,7	1,5

Scor = fen dominated by *Scorpidium cossonii* or *Hamatocaulis vernicosus*, Call = fen dominated by *Calliergonella cuspidata*, Sph = fen dominated by *Sphagnum palustre*.

The decrease in *E*_h_ during summer inundation to values of < 200 mV in the upper 10 cm in Scor-plots, reported to be representative for reduction of Fe(III) and SO_4_ [27], are in accordance with the idea of enhanced reduction processes. However, there were no indications for increased anaerobic decomposition, and enhanced Fe(III) and/or SO_4_ reduction in the form of changes in Fe-, S- or DOC-concentrations in porewater (S2 Fig). This may indicate that, despite of anaerobic circumstances, Fe(III) and SO_4_ reduction rates were still limited due to the fact that peat soils in the National Park Weerribben/Wieden are relatively low in redox-sensitive Fe and S [17].

The slight increase in internal P-mobilization during short-term summer inundation was not a cause for concern since porewater o-PO_4_ concentrations remained sufficiently low and did not threaten P-limited vegetation. NO_3_^-^ concentrations showed no considerable change either. NH_4_^+^ concentrations, however, showed a clear increase during summer inundation, which we attribute to increased infiltration rates of inundation water with relatively high NH_4_^+^ concentrations originating from the ditches. Maximum concentrations of 20 μmol L^-1^ under summer conditions are, however, not considered toxic to bryophyte vegetation or plants [28, 29].

### Effects for different vegetation types

Ca-concentrations, alkalinity and pH of inundation water in the Sph-plots were generally lower than in the Scor- and Call-plots. In addition, porewater ANC in the Sph-plots did not increase, even though infiltration occurred in all vegetation types and porewater ANC did increase in Scor- and Call-plots. The cation exchange capacity of the *Sphagnum*-comprising top layer of the Sph-peat soils has presumably hampered an increase in ANC via exchange of Ca^2+^ from inundation water for H^+^ [10, 12]. Moreover, pH values in inundation water at Sph-plots turned out lower during summer inundation than during winter inundation, which may indicate that the acidifying effect of *Sphagnum* is enhanced in summer, possibly as a result of increased growth rates. This points at the significant role of *Sphagnum* as an ecosystem engineer in changing its habitat under similar conditions, once the moss has invaded the vegetation.

### Conclusions and implications for management

In terms of counteracting acidification of rich fens, short-term summer inundation with base-rich surface water appears to be very efficient. In contrast to winter inundation, raising surface water levels in summer, when evapotranspiration rates are high, results in infiltration, and hence an increase of ANC. Secondly, internal alkalinity generation, as a result of anaerobic microbial redox processes, is enhanced by higher temperatures in summer. The latter effect will however be temporary, since aerobic oxidation during subsequent droughts can lead to re-acidification [16, 30]. The first process of infiltration of Ca-rich water, however, may contribute to a lasting increase in the peat soil ANC, as the ANC not only determined by the amount of bicarbonate in porewater in the circum-neutral pH range, but also by the saturation of Ca and Mg at the adsorption complex [14]. The ability of rich fen soils to exchange H^+^ for Ca^2+^ from the adsorption complex, and thereby buffer porewater pH, may be highly beneficial to counteract acidification during subsequent periods of drought in particular, when bicarbonate has been largely consumed and base cation exchange against H^+^ initiates.

Only in Scor- and Call-plots porewater ANC was increased by summer inundation. In Sph-plots, the ANC remained relatively low, presumably due to exchange of Ca^2+^ from inundation water for H^+^. Short-term summer inundation with base-rich water as a measure seems therefore only efficient at places where base-rich conditions still prevail. At the point when *Sphagnum* spp., which are able to acidify their environment, have already made their entry, the measure has no more effect. Therefore, short-term summer inundation is considered only a preventive measure, in order to maintain and restore current rich fens.

In addition to the importance of increased ANC to counteract acidification, raised water levels in summer may also be important to prevent other drought-induced problems that can be highly detrimental in rich fens. Increased oxygen availability during drought may lead to increased microbial decomposition, and hence increased mineralization of nutrients [16, 31], which can be highly detrimental for nutrient–limited rich fens. Furthermore, vegetation development and vitality of characteristic rich fen bryophytes are directly affected by drought in a negative way [17]. These adverse effects can be obviated as well by temporarily allowing raised water levels in summer.

Short-term summer inundation as a measure is, however, only considered beneficial under specific conditions. First, inundation has the most effect when the peat layer is attached to the underlying substrate via roots. In floating *Sphagnum*-dominated fens, raised surface water levels had almost no effect, because the buoyant peat follows changes in surface water levels and inundation does not occur, although this may be different in floating rich fens [12]. Further, surface water quality in adjacent ditches must not only be base-rich, but also nutrient-poor, as the adverse eutrophying effects of polluted inundation water on N- and P-limited vegetation are well-known [2]. In addition, porewater P-availability should not increase as a result of net P-mobilization (internal eutrophication) due to Fe(III) reduction in peat soils [19]. Especially in Fe-rich soils with high P-contents, this anaerobic P-mobilization can be severe [13, 17, 21]. Moreover, sulfate reduction and formation of FeS_x_ may result in additional P-mobilization [20, 32, 33, 34]. Finally, anaerobic conditions should not lead to formation of potential phytotoxins such as NH_4_^+^, H_2_S, Fe^2+^ and/or organic acids to plants, depending on soil chemistry [2, 17]. In the relatively Ca-rich fen sites of this study, both water and soil quality were suitable to obtain desired results from a management perspective. However, potential benefits and disadvantages of inundation need to be considered for different fen types with different water qualities separately in water management and nature management plans before implementation.

## Supporting Information

S1 Fig Redox potentials (*E*_h_) in the upper 20 cm of the soil in the three vegetation types during summer inundation in the KW-fen in 2013 and 2014. Scor (A) = fen dominated by *Hamatocaulis vernicosus*, Call (B) = fen dominated by *Calliergonella cuspidata*, and Sph (C) = fen dominated by *Sphagnum palustre*. The vertical white lines indicate the initiation and end of the treatment period. For interpolation, ordinary kriging was applied in ArcGIS (ArcMap 10.0, ESRI, Redlands, USA).
(PDF)

S2 Fig Fe (A), S (B), and DOC (C) concentrations per vegetation type in pore water 2 days before the experiment, in inundation water during the experiment, and in pore water 2 days after the experiment. Sample means with standard deviations are indicated (*n* = 5). Statistical information is provided in Table 3. For abbreviations see Table 1.
(PDF)
