# Supplementary material for: Short-Term Summer Inundation as a Measure to Counteract Acidification in Rich Fens
Source: PLoS One. 2015 Dec 4;10(12):e0144006. doi: 10.1371/journal.pone.0144006 (PMC4670166; doi:10.1371/journal.pone.0144006)
Supplement: S1 Fig — Scor (A) = fen dominated by Hamatocaulis vernicosus, Call (B) = fen dominated by Calliergonella cuspidata, and Sph (C) = fen dominated by Sphagnum palustre. The vertical white lines indicate the initiation and end of the treatment period. For interpolation, ordinary kriging was applied in ArcGIS (ArcMap 10.0, ESRI, Redlands, USA). (PDF) [file pone.0144006.s001.pdf]

Supplementary data in addition to:
Mettrop et al.: 'Short-term summer inundation as a measure to counteract acidification in rich fens' (PLOS ONE)

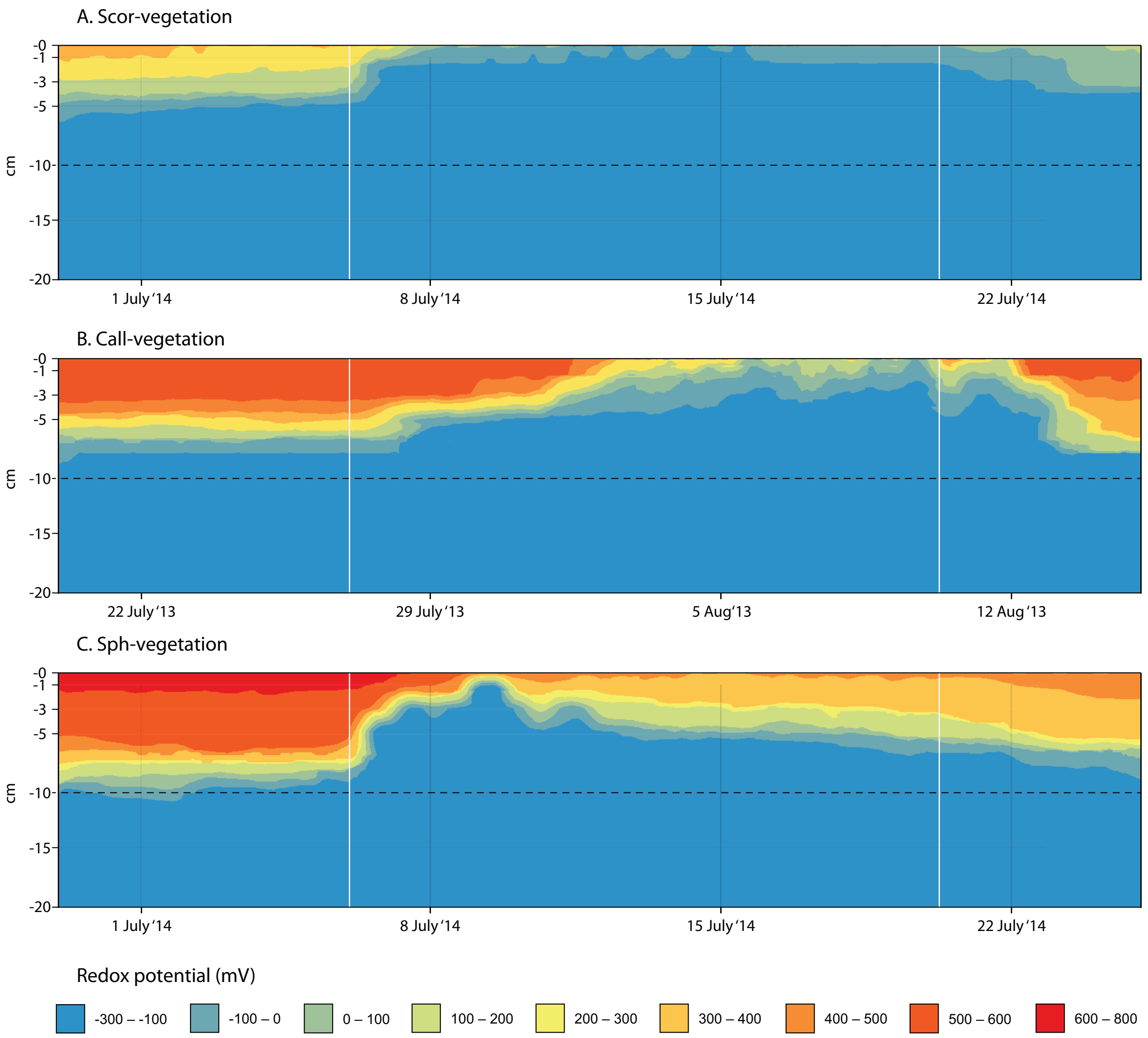

S1 Fig Redox potentials (E_h) in the upper 20 cm of the soil in the three vegetation types during summer inundation in the KW-fen in 2013 and 2014. Scor (A) = fen dominated by *Hamatocaulis vernicosus*, Call (B) = fen dominated by *Calliergonella cuspidata*, and Sph (C) = fen dominated by *Sphagnum palustre*. The vertical white lines indicate the initiation and end of the treatment period. For interpolation, ordinary kriging was applied in ArcGIS (ArcMap 10.0, ESRI, Redlands, USA).
