# Supplementary material for: Short-Term Summer Inundation as a Measure to Counteract Acidification in Rich Fens
Source: PLoS One. 2015 Dec 4;10(12):e0144006. doi: 10.1371/journal.pone.0144006 (PMC4670166; doi:10.1371/journal.pone.0144006)
Supplement: S2 Fig — Sample means with standard deviations are indicated (n = 5). Statistical information is provided in Table 3. For abbreviations see Table 1. (PDF) [file pone.0144006.s002.pdf]

Supplementary data in addition to:
Mettrop et al.: ‘Short-term summer inundation as a measure to counteract acidification in rich fens’ (PLOS ONE)

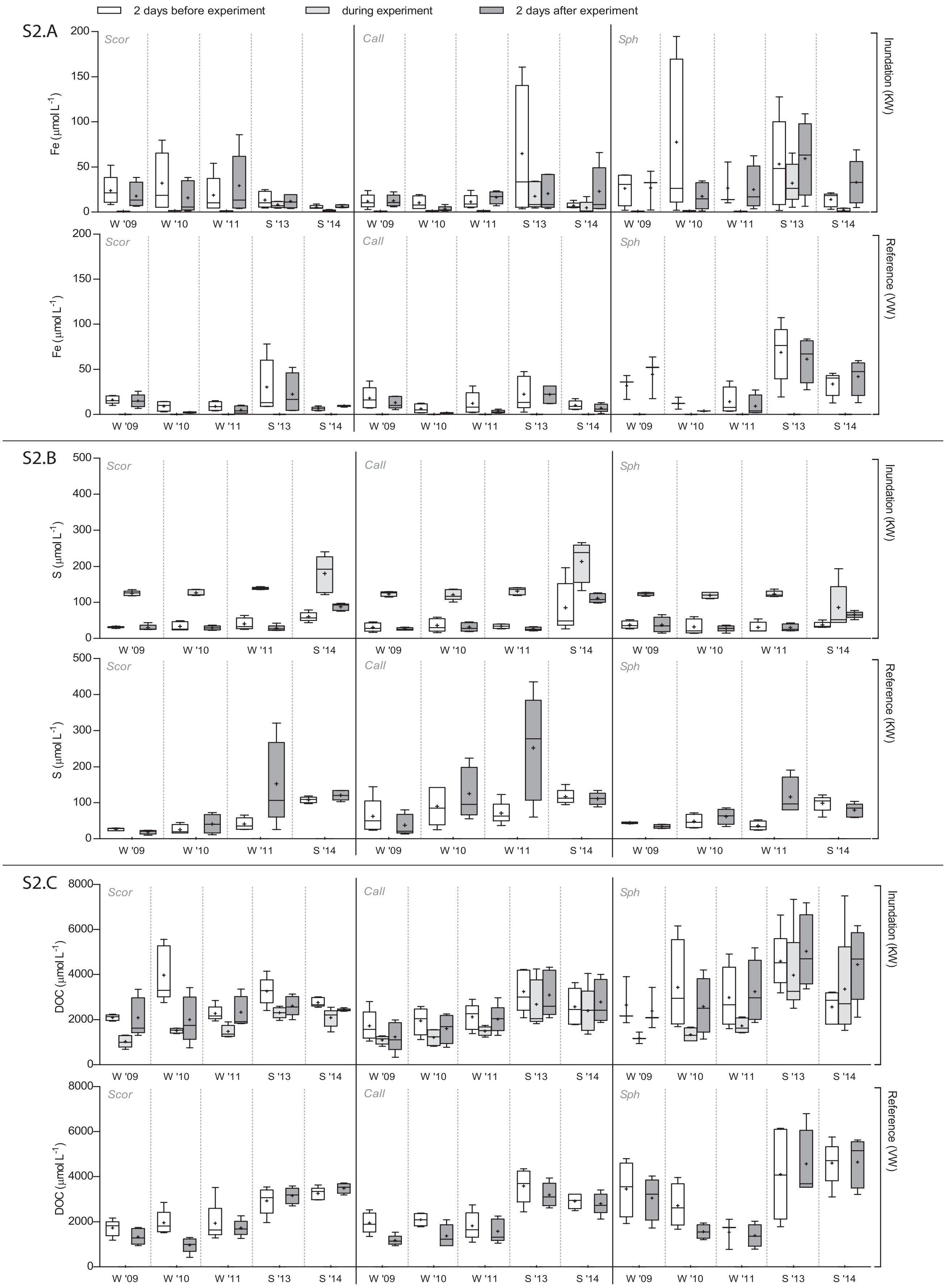

S2 Fig Fe (A), S (B), and DOC (C) concentrations per vegetation type in pore water 2 days before the experiment, in inundation water during the experiment, and in pore water 2 days after the experiment. Sample means with standard deviations are indicated (n = 5). Statistical information is provided in Table 3. For abbreviations see Table 1.
